# Bilateral carcinoid heart disease without intracardiac shunt in a patient with advanced functional small bowel neuroendocrine tumour: a clinical conundrum

**DOI:** 10.1093/ehjcr/ytaf679

**Published:** 2025-12-29

**Authors:** Stefano H Byer, Mashkurul Haque, Ola Abdelkarim, Christian Anderson, Udhayvir S Grewal

**Affiliations:** Department of Internal Medicine, University of Iowa Hospitals and Clinics, 200 Hawkins Dr, Iowa City, IA 52242, USA; Division of Cardiovascular Medicine, University of Iowa Hospitals and Clinics, 200 Hawkins Dr, Iowa City, IA 52242, USA; Department of Internal Medicine, University of Iowa Hospitals and Clinics, 200 Hawkins Dr, Iowa City, IA 52242, USA; Division of Cardiovascular Medicine, University of Iowa Hospitals and Clinics, 200 Hawkins Dr, Iowa City, IA 52242, USA; Division of Cardiovascular Medicine, University of Iowa Hospitals and Clinics, 200 Hawkins Dr, Iowa City, IA 52242, USA; Division of Cardiovascular Medicine, University of Iowa Hospitals and Clinics, 200 Hawkins Dr, Iowa City, IA 52242, USA; Division of Hematology, Oncology, and Blood and Marrow Transplantation, University of Iowa Hospitals and Clinics, 200 Hawkins Dr, Iowa City, IA 52242, USA; University of Iowa Neuroendocrine Tumor Program, Holden Comprehensive Cancer Center, 200 Hawkins Dr, Iowa City, IA 52242, USA

**Keywords:** Carcinoid heart disease, Neuroendocrine tumour, Intracardiac shunt, PRRT, Case report

## Abstract

**Background:**

Carcinoid heart disease (CHD) is a known complication of advanced functional neuroendocrine tumours (NETs), almost exclusively affecting right-sided cardiac valves. Left-sided involvement is rare and usually attributed to intracardiac shunting or pulmonary sources of serotonin. This case report highlights a rare presentation of CHD involving left- and right-sided valves in the absence of an anatomic shunt or bronchopulmonary NET.

**Case summary:**

A 67-year-old woman with a long-standing, functional, small bowel Grade 1 NET and metastatic liver and peritoneal disease presented with worsening dyspnoea and peripheral oedema. She had a 10-year disease history, previously managed with debulking surgery, somatostatin analogues, peptide receptor radionuclide therapy (PRRT), and everolimus. Echocardiography and cardiac magnetic resonance imaging demonstrated extensive left- and right-sided valvular involvement with severe mitral regurgitation, moderate aortic and pulmonic regurgitation, and mild tricuspid regurgitation, yet without intracardiac shunt. An elevated Qp/Qs ratio of 2.6 was attributed to severe left-sided valvular regurgitation. The patient improved on diuretic therapy and was referred for surgical evaluation of valve replacement prior to additional systemic treatment.

**Discussion:**

This case illustrates an atypical presentation of bilateral carcinoid valvulopathy in the absence of intracardiac shunting, likely due to overwhelming systemic serotonin from tumour burden. Although serotonin is typically inactivated in the lungs, extensive exposure may surpass this protective mechanism. The potential role of selective serotonin reuptake inhibitors remains inconclusive. Multidisciplinary coordination is essential for optimizing cardiac and oncologic outcomes, especially when systemic therapy such as PRRT is considered. Left- and right-sided CHD may develop in patients with small bowel NETs even without anatomic shunting. High tumour burden and systemic serotonin exposure may override pulmonary inactivation, leading to left-sided involvement. Early recognition and multidisciplinary care are critical for effective management.

Learning points

- Bilateral carcinoid heart disease can occur even in the absence of an intracardiac shunt, emphasizing that overwhelming systemic serotonin burden may bypass pulmonary inactivation.
- A high index of suspicion and multimodality imaging are crucial for diagnosing left-sided valvular involvement in patients with advanced functional neuroendocrine tumours.

## Introduction

Carcinoid heart disease (CHD) is a well-recognized complication of advanced functional neuroendocrine tumours (NETs), mostly of small bowel origin. Carcinoid heart disease encompasses a constellation of clinical signs and symptoms associated with cardiac dysfunction that results from valvular damage owing to excessive production of serotonin and other vasoactive substances. Carcinoid heart disease typically impacts the right side of the heart due to the protective inactivation of serotonin and other biogenic amines in the lungs; however, left-sided and even bilateral involvement may be seen in some circumstances.^1,2^ Notably, bilateral involvement is seen in the presence of an intracardiac shunt, such as a patient foramen ovale or an atrial/ventricular septal defect.^3^ Bilateral involvement may also be observed in functional bronchopulmonary or thymic NETs due to circulation of serotonin in both the right- and left-sided circulation of the heart.^4^ Conflicting evidence also suggests a potential role of selective serotonin reuptake inhibitors (SSRIs).^5,6^ Here, we discuss a rare case of a patient long-standing small bowel NET complicated by progressive left- and right-sided carcinoid valvulopathy in the absence of intracardiac shunt and discuss key diagnostic, therapeutic, and multidisciplinary considerations.

## Case summary

A 67-year-old woman with a well-differentiated (WHO grade 1, Ki-67 <2%) functional small bowel NET presented with worsening shortness of breath and bipedal oedema. She also had long-standing history of major depressive disorder on therapy with citalopram. Prior to this, she had a 10-year history since her initial diagnosis of functional Grade 1 NET (intermittent flushing and diarrhoea) with *de novo* metastatic disease (peritoneal and liver metastases) and was diagnosed in 2015 with peritoneal and liver metastases. Subsequently, she underwent cytoreductive debulking surgery with resection of the primary tumour and cholecystectomy after which she was initiated on long-term somatostatin analogue (SSA) therapy with Sandostatin LAR. On subsequent disease progression, she underwent treatment with ¹⁷⁷Lu-DOTATATE peptide receptor radionuclide therapy (PRRT) which was followed by prolonged disease stability. More recently, she was initiated on therapy with everolimus due to both clinical and radiographic disease progression; however, this had to be discontinued due to recurrent Grade 3 transaminitis.

Upon current presentation, the patient was admitted for inpatient evaluation due to new-onset exertional dyspnoea and bilateral lower extremity oedema, raising concern for possible heart failure. Her vital signs were a blood pressure of 180/54 mmHg, pulse 74 b.p.m., afebrile temperature 36 °C, respiratory rate 16/min, and SpO₂ 97% on room air; bedside examination showed elevated JVP (∼10 cm), diminished breath sounds without rales, a Grade 3/6 systolic murmur at the left sternal border and apex, and bilateral lower-extremity oedema.

Laboratory testing revealed an elevated NT-proBNP level of 4596 pg/mL (reference range < 125 pg/mL) with WBC 7.5 × 10³/, haemoglobin 9.0 g/dL, creatinine 1.20 mg/dL on admission, alkaline phosphatase 188 U/L, and INR 1.0. Of note, C-reactive protein was note collected. The 12-lead electrocardiogram showed sinus rhythm.

Chest radiography showed a normal cardiomediastinal silhouette with focal right perihilar peribronchial thickening with ground-glass opacity and a small right pleural effusion, with a clear left lung; computed tomography angiogram chest demonstrated no pulmonary emboli, enlarged main pulmonary artery (3.4 cm), biatrial and LV dilatation with reflux into hepatic veins, and interval increase in loculated right pleural effusion with pleural metastases and adjacent atelectasis.

Transthoracic echocardiography (TTE) demonstrated hallmark features of carcinoid valvulopathy. The mitral valve leaflets were mildly thickened with severe mitral regurgitation [effective regurgitant orifice area (EROA) 0.41 cm²], and the aortic valve was trileaflet with moderate-to-severe regurgitation. The pulmonic valve showed moderate regurgitation, while the tricuspid valve appeared morphologically normal with mild regurgitation. Left ventricular size and systolic function were normal [LVEF 57% by biplane Simpson’s method; global longitudinal strain −22.5% (normal range: −18% to −30%)], and the right ventricle was normal in size and function. Estimated pulmonary artery systolic pressure was ≈45 mmHg based on a TR peak gradient 42 mmHg. There was left atrial enlargement (5.47 cm), normal right atrial size, and a small pericardial effusion with trace right-sided pleural effusion. Further, LVIDd 5.47 cm, LVIDs 3.52 cm, LVEF 55%–60%, severely enlarged left atrium, normal right-sided chambers, trace tricuspid regurgitation, moderate-to-severe aortic insufficiency, and estimated PASP 30–40 mmHg were among other notable findings. Transthoracic echocardiography 3 years prior had described only mild aortic insufficiency and trace valvular disease along with LVIDd 4.0 cm andLVIDs 2.7 cm, demonstrating interval progression (*Table 1*).

**Table 1:** Comparative echocardiographic parameters from prior and current TTE with reference ranges

Parameter	Prior TTE (2022)	Admission TTE (2025)	Unit	Reference range (normal limits)
LV dimensions				
LVIDd	4.0	5.5	cm	3.9–5.3
LVIDs	2.7	3.74	cm	2.3–3.9
LV systolic function				
LVEF	Normal (qualitative)	57	%	≥55
GLS	—	−22.5	%	−18 to −30
Atrial size				
LA size	—	5.47 (enlarged)	cm	≤4.0
RA size	—	Normal	cm	≤4.4
Right-sided pressures				
TR gradient	—	42	mmHg	≤30
PASP	—	45	mmHg	≤35
Valvular lesions				
MR EROA	—	0.41 (severe)	cm²	<0.20
AR severity	Mild	Moderate–severe	—	None–mild
PR severity	—	Moderate	—	None–mild

The table summarizes key chamber dimensions and valvular haemodynamics demonstrating progression of carcinoid valvulopathy. Between the prior TTE (2022) and the current admission study (2025), there was an increase in left ventricular diastolic dimension (LVIDd 4.0 → 5.5 cm) and severe left atrial enlargement (5.47 cm), while systolic function remained preserved (LVEF 57%, GLS −22.5%). New or worsened multivalvular regurgitation was evident, including severe mitral regurgitation (EROA 0.41 cm²), moderate–severe aortic regurgitation, and moderate pulmonic regurgitation, with elevated tricuspid gradient (42 mmHg) and pulmonary artery systolic pressure (45 mmHg). Reference ranges are derived from American Society of Echocardiography (ASE) chamber quantification and valvular guidelines.

Transoesophageal echocardiography (TEE) revealed severe mitral regurgitation (PISA EORA 0.6 cm²) with three regurgitant jets and flow reversal in the pulmonary veins, moderate tricuspid regurgitation, moderate-to-severe aortic insufficiency, and moderate pulmonic regurgitation. The left ventricular systolic function was preserved, and the RV/RA peak systolic gradient was 46 mmHg. No interatrial shunt was visualized by colour Doppler, and no right-to-left shunt was detected on agitated-saline contrast. The left atrial appendage was free of thrombus. Findings were consistent with biventricular carcinoid valvulopathy in the absence of an anatomic shunt.

**Figure 1:**
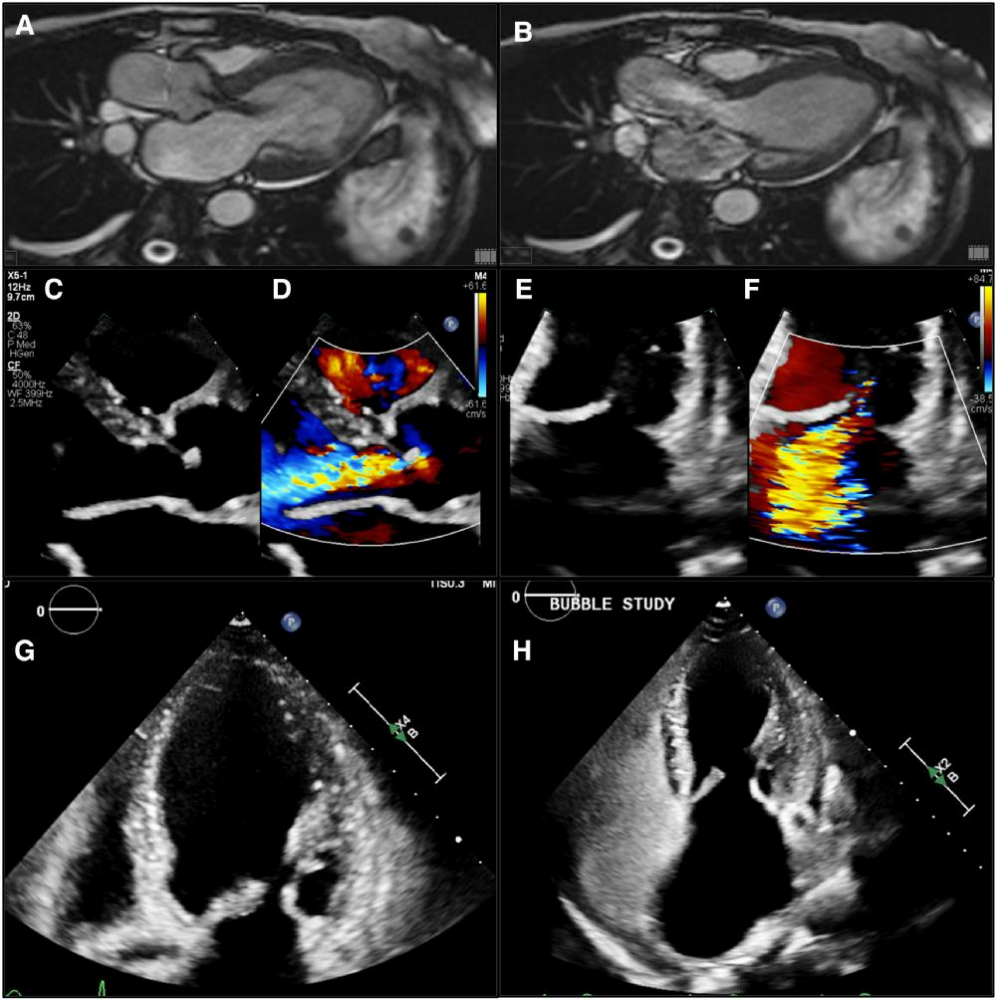
Multimodality imaging findings in a patient with bilateral carcinoid heart disease in the absence of an intracardiac shunt. Panel (*A*) shows a cardiac magnetic resonance three-chamber steady-state free precession cine revealing aortic regurgitation and a thickened anterior mitral valve leaflet, while panel (*B*) demonstrates severe mitral regurgitation on the same view. Panels (*C*) and (*D*) display transthoracic echocardiogram images highlighting the mitral valve morphology with thickened, retracted leaflets and colour Doppler confirming severe mitral regurgitation, respectively. Panel (*E*) shows the transthoracic echocardiogram image of the aortic valve morphology, and panel (*F*) depicts colour Doppler evidence of moderate aortic insufficiency. The apical four-chamber view in panel (*G*) further demonstrates thickened mitral valve leaflets. Finally, panel (*H*) presents an agitated saline contrast study confirming the absence of a right-to-left shunt.

To further delineate the underlying cardiac pathology and investigate the presence of an intracardiac shunt, a cardiac magnetic resonance imaging (MRI) was performed. This further evidenced underlying biventricular CHD, with no evidence of delayed enhancement or myocardial scar. The MRI highlighted moderate mitral regurgitation, moderate aortic regurgitation, and mild-to-moderate pulmonic regurgitation, but no intracardiac shunt. Interestingly, the pulmonary-to-systemic flow ratio (Qp/Qs) was elevated at 2.6, attributed to significant left-sided valvular regurgitation, supporting the diagnosis of left-sided involvement despite the absence of an anatomic shunt. These findings confirmed significant carcinoid valvulopathy affecting both sides of the heart (*Figure 1*).

The patient was initiated on daily diuretic therapy (intravenous followed by oral furosemide), which allowed notable improvement in dyspnoea and lower extremity oedema. The case was reviewed at our institutional multidisciplinary tumour board. Given the extensive bilateral valvular involvement and risk of carcinoid crisis, evaluation by cardiothoracic surgery to assess candidacy for potential valve replacement or repair before considering repeat PRRT was recommended. She remains under close outpatient monitoring by our multidisciplinary team including cardiology, medical oncology, and cardiac surgery, with a focus on optimizing her cardiac status while managing her progressive metastatic functional NET.

## Discussion

This case highlights the unique challenges posed by bilateral CHD. In our differential diagnosis of multivalvular regurgitation we considered CHD, degenerative (myxomatous) mitral valve disease, rheumatic valvulitis, infective endocarditis, drug-induced valvulopathy (ergot/fenfluramine), radiation-associated valvular disease, autoimmune valvulitis, and shunt-mediated left-sided involvement. Absence of fever, normal leukocyte count at presentation, and no vegetations on TEE argued against endocarditis; there was no history of mediastinal radiation or ergot exposure. Transoesophageal echocardiography (with agitated saline) and CMR showed no intracardiac shunt, and imaging did not reveal a bronchopulmonary NET. With long-standing functional small-bowel NET and typical valvular morphology (thickened/retracted leaflets with predominant regurgitation), the overall picture favoured bilateral carcinoid valvulopathy. The elevated CMR-derived Qp/Qs (2.6) reflects regurgitant volume recirculation in severe MR with coexistent AR: regurgitant flow augments pulmonary venous return (Qp) while forward systemic stroke volume (Qs) is reduced by regurgitation, yielding Qp greater than Qs despite the absence of a shunt.

Classical CHD typically affects right-sided valves owing to the inactivation of serotonin and other vasoactive substances in the lung.^1,2^ Left-sided valvular involvement is uncommon, seen in approximately 20% of cases, and typically suggests either a right-to-left shunt or massive serotonin levels that overwhelm pulmonary inactivation.^7^ In the current patient, the absence of an intracardiac shunt on imaging underscores the possibility of ‘serotonin spillover’ due to extensive tumour burden, particularly in the setting of progressive metastatic disease.

Carcinoid valvulopathy results from serotonin-mediated activation of 5-HT₂B receptors on valvular interstitial cells, promoting fibrous plaque deposition and leaflet fixation. This process leads to regurgitant rather than stenotic lesions.^1^ The impact of carcinoid valvulopathy on cardiac function can be insidious but devastating, culminating in right or left heart failure, depending on the valves involved. Notably, patients with CHD have an approximately two-fold higher mortality than those without cardiac involvement (69% vs. 31%).^8^ Clinically, the presence of left-sided disease may portend an even poorer prognosis, given the severity of cardiac dysfunction and the extent of systemic hormone exposure and tumour burden that may underpin the development of bilateral CHD.

The data implicating the role of SSRIs in the development of CHD are conflicting. A prior report described the case of a patient who was on treatment with an SSRI and developed bilateral CHD in the setting of controlled carcinoid syndrome symptoms. The authors hypothesized that a circulatory overload that potentially overwhelmed the capacity of the lung to inactivate and metabolize serotonin may have contributed to the development of bilateral CHD.^5^ However, a retrospective analysis of 92 patients with advanced NETs did not demonstrate an association between SSRI use and the risk of CHD.^6^ Of note, a pilot study also noted similar levels of serotonin in right- and left-sided circulation in patients with CHD, hypothesizing the role of vasoactive substances other than serotonin in the pathogenesis of CHD.^9^

Among patients with CHD, achieving disease control is critical to slowing valvular damage. Peptide receptor radionuclide therapy has emerged as an effective option for disease and symptom control, but its use in patients with advanced CHD warrants some key considerations. Infusion of amino acids during PRRT for renal protection along with intravenous fluids can exacerbate fluid overload, and the risk of carcinoid crisis during therapy is heightened in patients with uncontrolled hormone secretion or severe valvular dysfunction.^10^ Valve replacement surgery is often necessary in severe cases, particularly when symptoms persist despite medical therapy and the patient’s anticipated survival exceeds one year.^11^ In advanced CHD with refractory symptoms, valve surgery at experienced centres is associated with meaningful symptomatic improvement and acceptable early risk, supporting consideration when anticipated survival exceeds ∼1 year. The patient is under consideration for surgical valve replacement prior to PRRT, as of this writing.

The current case highlights the possibility of the development of left-sided carcinoid valvulopathy even in the absence of an intracardiac shunt or underlying bronchopulmonary carcinoid tumour. While predominantly right-sided cardiac involvement remains the hallmark of CHD due to pulmonary inactivation of serotonin, excessive systemic hormone burden may overwhelm this protective mechanism, resulting in biventricular involvement. Future prospective studies are needed to better understand the safety and cardiovascular implications of SSRIs in patients with advanced functional NETs. A coordinated, multidisciplinary approach is essential to balance disease control with cardiac risk management among patients with CHD.

## Lead author biography


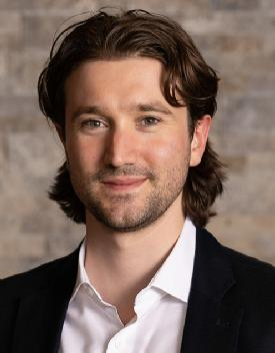


Stefano H. Byer, MD, MS, is a physician and clinical researcher focused on cardiovascular medicine, cardio-oncology, and advanced heart failure. His work spans outcomes research using large real-world datasets, innovation in digital health and patient-centred technologies, and the study of cardiotoxicity in cancer therapies, amyloidosis, and mechanical circulatory support. He has published across leading cardiology and oncology journals and collaborates with national experts to advance precision care for complex cardiac patients. Dr Byer is also committed to medical education, mentorship, and translational innovation at the intersection of cardiology, oncology, and emerging technologies.

## Supplementary Material

ytaf679_Supplementary_Data

## Data Availability

The data underlying this article will be shared on reasonable request to the corresponding author.

## Supplementary material

**Consent:** The authors confirm that witnessed verbal consent for submission and publication of this case report including images and associated text has been obtained from the patient. This has been discussed with the editors.
