# Supplementary material for: Bilateral carcinoid heart disease without intracardiac shunt in a patient with advanced functional small bowel neuroendocrine tumour: a clinical conundrum
Source: Eur Heart J Case Rep. 2025 Dec 29;10(1):ytaf679. doi: 10.1093/ehjcr/ytaf679 (PMC12836417; doi:10.1093/ehjcr/ytaf679)
Supplement: ytaf679_Supplementary_Data [file ytaf679_supplementary_data.pdf]

European Heart Journal - Case Reports

Lack of written consent form

This form should be used by authors wishing to submit a case report/case series/grand round/images in cardiology article to *European Heart Journal – Case Reports* where written consent is not available for a patient included in the manuscript.

The *Editorial Board* of *European Heart Journal – Case Reports* believes that patients should consent to the publication of their cases. However, it is also appreciated that there are some circumstances where the ability to gain informed consent is not possible or appropriate.

Please use the lack of written consent flow chart to confirm the action that is required. This form should only be used as outlined in this flowchart.

Manuscript Title: Bilateral Carcinoid Heart Disease in Advanced Functional Small Bowel Neuroendocrine Tumor Without Intracardiac Shunt: A Clinical Conundrum

Manuscript ID (if known): ………

Scenario A – Witnessed verbal consent to publish has been obtained from the patient but written consent is not possible

A.1. Please outline the reason(s) why written consent from the patient has not been possible in this situation:

Due to logistic challenges, the patient was unable to provide a written consent. She lives far away from our tertiary referral institution and provided verbal consent for inclusion of her case in this report.

A.2 Who witnessed the verbal consent **Mashkurul Haque, MD**

A.3 *If possible, please provide the date of the witnessed consent…* **July 10, 2025**

……

Scenario B – Verbal consent to publish has been obtained from the patient but not witnessed and written consent is not possible

B.1. Please outline the reason(s) why written consent from the patient has not been possible in this situation:

……

……

……

B.2. Please outline the reason(s) why witnessed verbal consent from the patient has not been possible in this situation:

……

……

……

Scenario C – The patient is deceased and while there are next-of-kin/surviving relatives, it is not possible to contact them.

C.1. Please outline the reason(s) why the next-of-kin/surviving relative(s) cannot be contacted in this situation:

………

………

………

C.2. If the next-of-kin/surviving relatives contact details are not available, please outline what attempts have been made to obtain these details:

………

………

………

Scenario D – The patient is deceased and has no surviving relatives / next-of-kin.

D.1. Please provide details of this scenario and how it has been confirmed that there are no appropriate surviving relatives to contact:

………

………

………

Scenario E – The patient is alive but has not been contacted to seek consent to publish due to legal or ethical restrictions.

E.1. Please provide details of the legal and/or ethical restrictions that prevent contact with the patient to seek consent to publish:

………

………

………

Scenario F – The patient is alive but has not been contacted to seek consent to publish and there are no contact details for the patient.

F.1. Please provide details of the steps taken by the authors to obtain contact details for the patient:

………

………
